# Key Predictors of Treatment Failure in Conservatively Managed Spondylodiscitis: A Long-Term Retrospective Cohort Study

**DOI:** 10.3390/jcm14061973

**Published:** 2025-03-14

**Authors:** Fatma Kilinc, Matthias Setzer, Florian Gessler, Vincent Prinz, Daniel Jussen, Marcus Czabanka, Thomas Freiman, Bedjan Behmanesh

**Affiliations:** 1 Department of Neurosurgery, Goethe University Hospital, Schleusenweg 2-16, 60528 Frankfurt am Main, Germany; matthias.setzer@unimedizin-ffm.de (M.S.); vincent.prinz@unimedizin-ffm.de (V.P.); daniel.jussen@unimedizin-ffm.de (D.J.); marcus.czabanka@unimedizin-ffm.de (M.C.); 2 Department of Neurosurgery, University Medicine of Rostock, 18057 Rostock, Germany; florian.gessler@med.uni-rostock.de (F.G.); thomas.freiman@med.uni-rostock.de (T.F.); bedjan.behmanesh@med.uni-rostock.de (B.B.)

**Keywords:** spondylodiscitis, conservative therapy, osteolysis, outcome

## Abstract

**Background/Objectives**: Conservative treatment of patients with pyogenic spinal infection is a well-established therapy. Nevertheless, treatment failure is often seen despite adequate antibiotic therapy. The aim of this study was to evaluate predictors of treatment failure facilitating individualized, patient-specific therapy and thus increasing the success of therapy. **Methods**: We retrospectively evaluated medical data and radiological findings of patients who were admitted between 2012 and 2023. Treatment failure and readmission were analyzed. Vertebral body lesions and spinal deformity were assessed at admission and follow-up. Patient comorbidities were assessed using the Charlson Comorbidity Index (CCI). Each patient underwent CT and MR imaging of the affected part of the spine. During follow-up, a new CT scan was performed to show the extent of the spinal lesion. **Results**: A total of 245 patients with a mean age of 65.4 years were included in the final analysis. The gender distribution included 164 (67%) male and 81 (33%) female patients. The mean follow-up time was 46 months (range 5–96 months). Among 245 patients, 86 (35.1%) received conservative therapy, and treatment failure was observed in 34 (40%) of them, compared to 6 (4%) in the surgical group (*p* < 0.001). The progression of vertebral body lesions was identified as a potential reason for treatment failure in these patients. Radiological follow-up data revealed an average of 32% destruction compared to the initial CT scan. A significant association was observed between treatment failure and age (*p* = 0.007, CI 95%: 97.8–100%), cervical discitis (*p* = 0.018, CI 95%: 97.3–100%) and high CCI scores (*p* = 0.001, CI 95%: 98.3–99.5%). **Conclusions**: In our study, we were able to show that factors such as age, position of the cervical spine and a high CC index were significantly associated with treatment failure. This was due to increasing bone destruction. Early surgical treatment may prevent the progression of osteolysis and long-term complications such as persistent back pain and the need for subsequent complex surgery. These predictors may also help guide individualized, patient-specific therapy for conservatively treated patients, thereby improving treatment outcomes.

## 1. Introduction

Spondylodiscitis is the most common spinal infection, affecting the intervertebral disk, adjacent vertebral bodies and in some cases, the posterior elements of the spine [1,2,3,4]. The incidence of spondylodiscitis in Western countries has been reported to range from 0.2% to 2.4% per 100,000 per year [4,5]. This increasing incidence is attributed to an aging population with increasing comorbidities, as well as increased life expectancy and improved imaging techniques and clinical diagnostics [1,2,6]. Despite advances in diagnostic and therapeutic options in recent decades, spondylodiscitis still has a high mortality rate of 2–20% [7,8,9,10].

Although some treatment guidelines exist, there is no universally accepted standard for spondylodiscitis management, and treatment approaches vary based on local clinical practices. At present, conservative treatment, in patients without neurological deficit, minimal bony destruction and absence of a spinal epidural abscess, is the most commonly used treatment option. It is also often the first choice in elderly patients and those in poor general health, especially given the risks of surgery [9,10]. However, conservative treatment carries potential risks of failure, including progression of osteolysis, chronic pain syndromes, residual neurologic deficits and systemic inflammation/infection, which may ultimately necessitate surgical intervention. Persistent pain, especially in elderly patients, can also lead to increasing immobility, which can favor complications such as deep vein thrombosis, pulmonary embolism and pneumonia. In addition to these risks, the rates of pseudarthrosis and instability, which can lead to kyphotic deformities and chronic pain syndromes, are relatively high at 16–50% [9,11,12,13,14]. The most common indication for surgery is the absence of the above factors and the presence or worsening of a neurological deficit followed by failure of conservative management with antibiotics [15]. These can be associated with a more complex surgery. Therefore, the aim of this study is to identify factors that may serve as predictors of treatment failure in conservatively treated patients, with the aim of facilitating individualized, patient-specific therapy and thus increasing the success of therapy.

## 2. Methods

**Figure 1:**
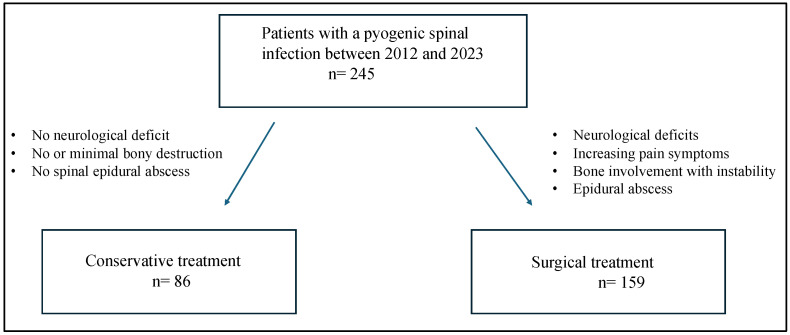
Flowchart describing the management of patients with pyogenic spinal infection.

In this retrospective study, we analyzed the clinical and radiological data of a total of 245 patients being admitted with a pyogenic spinal infection between 2012 and 2023. Inclusion criteria were patients over 18 years of age with a first diagnosis of pyogenic or tuberculous spondylodiscitis. Exclusion criteria were incomplete medical records or imaging data and patients with missing outpatient follow-up (Figure 1). All included patients were divided into two groups, conservatively versus operatively. The location of infection was divided into cervical and cervicothoracic (C1-Th2), thoracic (Th3-Th10), thoracolumbar (Th11-L2) and lumbar and lumbosacral (L3-S2).

Patient information, including characteristics at admission and during treatment, laboratory values, presenting symptoms and functional neurological status at admission, were recorded for all included patients. Patient comorbidities were assessed using the Charlson Comorbidity Index (CCI).

Conservative treatment was advocated in patients without neurological deficit, minimal bony destruction and absence of a spinal epidural abscess. Surgical intervention was performed when neurological deficits, increasing pain symptoms, bone involvement with instability and/or epidural abscess existed. Surgical treatment consisted of instrumented stabilization in conjunction with debridement and graft interposition.

To confirm spondylodiscitis, all patients underwent CT, MRI and blood cultures. For conservatively treated patients, microbiological results were obtained from blood cultures and/or CT-guided biopsy. Antibiotic susceptibility testing was performed, and an appropriate bactericidal agent was administered.

For operatively treated patients, microbiological results were obtained intraoperatively. Specific antibiotic therapy consisted of intravenous application for 2 weeks and oral administration for a further 6 weeks.

Follow-up MRI and CT scans were performed postoperatively and at least 3 months after treatment in both groups to evaluate the success of the treatment. After surgery, spinal fusion was analyzed on the basis of the follow-up CT scan and additionally assessed by a radiologist. In patients who received conservative treatment only, healing was analyzed using CT and MRI scans. Inflammatory parameters were also measured in all patients. They were also asked about symptoms such as pain.

Treatment failure was defined as readmission because of pain progression, developing new neurological deficits and/or progressive osteolysis despite antibiotic therapy. In these cases, new CT and MRI scans were performed after readmission.

**Figure 2:**
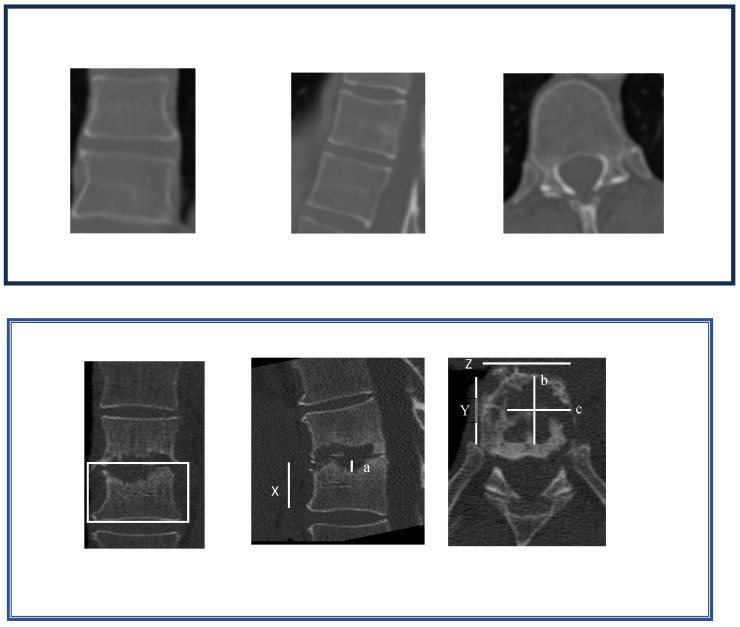
Method to estimate the extent of osteolysis.

To evaluate the vertebral structure, the vertebral body volume as well as the extent of destruction were estimated by measuring their maximum sizes on a CT scan at admission, after therapy and in cases of readmission in each of the three orthogonal planes (X, Y, Z). Height and width were measured on the frontal reconstruction (a, c), and the depth was measured on the horizontal reconstruction (b) [14]. The percentage of osteolysis (PO) was calculated as follows (Figure 2 shows a comparison of the bones before and after antibiotic treatment):

$$PO=\frac{a\cdot b\cdot c}{X\cdot Y\cdot Z}$$

Long-term follow-up and complications after conservative and surgical treatment were recorded and analyzed. For this purpose, a CT scan was performed to detect any increased osteolysis.

### 2.1. Statistical Analysis

Statistical comparisons were performed using chi-square tests for categorical variables and independent *t*-tests for continuous variables. Logistic regression analysis was used to identify independent predictors of treatment failure. A *p*-value < 0.05 was considered statistically significant.

### 2.2. Data Availability

The datasets generated during and/or analyzed during the current study are available from the corresponding author on reasonable request.

## 3. Results

Ultimately, 245 patients with a mean age of 65.4 years were included for final analysis. The sex distribution included 164 (66.9%) male and 81 (33.1%) female patients. In 86 (35.1%) cases, patients were first treated conservatively and 156 patients underwent surgery. In the conservative group, 34 (40%) patients underwent secondary surgery. There were six (3.8%) cases of treatment failure in the surgical group.

In the conservative group, spondylodiscitis was limited to a single level in 75 cases (88.3%), with the lumbosacral region being the most common location of infection (60.5%). In the surgical group, 108 cases (67.9%) were also limited to a single level and the most common location of infection was the lumbosacral region.

Patients’ comorbidities were assessed according to the CCI. In the conservatively treated cohort, 24.4% had a CCI of 4, followed by 5 (38.4). In 14 cases (16.3), the CCI index was 3 and in 10 cases (11.6), it was 2. A total of eight cases had a CCI of 0 or 1. In the operative-treated cohort, in most cases, there was a CCI of 4 (29.6) followed by a CCI of 3 (25.8). A CCI of 5 was present in 21 cases (13.2). In 28 cases, patients had a CCI of 2 (17.6) and in a total of 22 cases, a CCI of 0 or 1 (4.4 vs. 9.4). The dominant symptom leading to admission in both groups was back pain (98.8%) (Table 1 and Table 2).

The microbiology results were based on blood cultures, CT-guided biopsy or due to intraoperative tissue. The most common offending organism for the whole cohort was coagulase-positive staphylococcus aureus, followed by other staphylococcus species and other coagulase-negative streptococci. In cases of the identification of microorganisms, a bactericidal and resistogram antibiotic was administered. For the conservative cohort in 9 cases (10.5) and for the operatively treated cohort in 18 cases (11.3), the microbiology results were negative. In these cases, an infectious disease team was consulted prior to antimicrobial therapy. In a total of 15 cases, intravenous therapy with vancomycin and meropenem for 2 weeks was recommended. In a total of 12 cases, intravenous ampicillin/sulbactam was recommended instead of vancomycin and meropenem because of renal insufficiency. For all 27 patients, the recommendation was to continue oral sequential therapy with rifampicin and levofloxacin for a further 6 weeks (Table 3 and Table 4).

### 3.1. Treatment Failure

#### 3.1.1. Conservatively

There was a 40% treatment failure in conservatively managed patients compared to 4% in surgically treated patients, *p* ≤ 0.001. From 34 patients with readmission due to treatment failure, pain progression was the most common symptom and was reported by 30 patients; 4 patients additionally developed a new neurological deficit. CT and MRI scans were performed after readmission. Finally, in 28 cases (82.4), a CT scan showed an increase in osteolysis. The average of vertebral body destruction was 31.9% with consecutive kyphosis compared to the initial scan in conservatively managed patients. Laboratory tests also showed an increase in inflammatory parameters in 37.5% of patients despite antibiotic treatment. Thirty patients (88.2%) underwent surgery, while the remaining four refused surgery due to lack of neurological symptoms. Of these 34 patients, 10 were found to have Staph aureus. A different Staphylococcus species was found in 11 patients, while no bacteria could be detected in the remaining 13 patients.

No multidrug-resistant bacteria were found in these patients. These patients were treated with antibiotics according to the antibiogram. All patients confirmed that they had taken their antibiotics regularly.

#### 3.1.2. Operatively

In addition, 6 (4) out of 159 patients undergoing surgical treatment revealed a treatment failure as well and were readmitted due to pain progression in 4 cases and new neurological deficit in 2 cases. In contrast, the amount of vertebral body destruction in the surgically treated cohort was an average of 3.7%. A deformity was not observed. It was found that the antibiotic therapy was not taken consistently in four cases. The inserted material was surgically replaced and a new debridement was performed.

Direct surgical treatment was performed in 159 (65%) cases, with an observed increase in osteolysis postoperatively in 6 cases, averaging 3.8%. Two patients experienced progressive neurological deficits and four had intolerable pain due to material fracture and instability requiring surgery. However, after surgery, there was no significant increase in bone osteolysis in either the conservative or surgical groups (Table 5).

A significant association was observed between treatment failure and age (*p* = 0.007, 95% 97.8–100%), cervical discitis (*p* = 0.018, CI 95% 97.3–100%) and high CCI scores (*p* = 0.001, CI 95% 98.3–99.5%) (Table 6).

### 3.2. Follow-Up

At the end of the follow-up period, the outcomes for 198 patients were evaluated (128 operative vs. 52 conservative). The 34 secondary operatively treated patients were also included in these 128 patients. A total of 65 of the operatively treated patients could not be contacted. The mean follow-up time was 37.1 ± 31.8 (range 6–96 months).

In the operative cohort, all included patients had a good outcome and demonstrated clinical healing of the spinal infection. The patients no longer described back pain. Only in 28 cases (21.9%) did patients describe intermittent back pain that does not restrict their everyday life. The laboratory results were unremarkable. There was no evidence of infection. Bony fusion was achieved in 97.7% of cases, with no evidence of loosening or breakage of internal fixation. In three cases, the fixation was removed at the patient’s request after healing of the infection. In the follow-up CT scan, no deformity was present.

The 30 patients who underwent initial conservative treatment followed by surgery were very satisfied with the clinical outcome. A total of 12.5% reported mild back pain that did not interfere with daily activities. CT showed no more deformity or increase in osteolysis.

The only conservative-treated group, without secondary surgery, (52 patients) also demonstrated clinical healing of the spinal infection. This cohort was only treated conservatively. Secondary surgery was not performed. Nevertheless, from these 52 patients, 33 (63%) described residual back pain. For 14 (42.4) of these 33 patients, the CT scan showed the onset of kyphosis. The initial CT scan, which was performed at the time of diagnosis, showed no deformity.

## 4. Discussion

Spondylodiscitis remains a life-threatening disease with a high mortality rate of 2–20% [8,9,10,16]. In order to achieve a favorable functional outcome, as well as chronic pain, early diagnosis is crucial. As reported in the literature, a delayed diagnosis is associated with a poor outcome [17,18]. Untreated vertebral infections may progress to secondary instability that may develop towards kyphosis deformity with paraplegia or other neurological deficits. Despite the existence of some therapeutic guidelines, the treatment of spondylodiscitis is certainly not standardized and is mostly based on local preferences, which leads to variability among physicians [12,16]. Conservative treatment, consisting of prolonged antibiotic therapy, appears to be the treatment of choice for the majority of patients [7,19].

In our study, the microbiology results were based on blood cultures, CT-guided biopsy or due to intraoperative tissue. The most common offending organism for the whole cohort was coagulase-positive staphylococcus aureus, followed by other staphylococcus species and other coagulase-negative streptococci. In cases of the identification of microorganisms, a bactericidal and resistogram antibiotic was administered. For the conservative cohort in 10.5% and for the operatively treated cohort in 11.3%, the microbiology results were negative. In these cases, an infectious disease team was consulted prior to antimicrobial therapy. Intravenous therapy with vancomycin and meropenem for 2 weeks was recommended. In some cases, intravenous ampicillin/sulbactam was recommended instead of vancomycin and meropenem because of renal insufficiency. The recommendation for oral sequential therapy was rifampicin and levofloxacin for a further 6 weeks.

The microbiological profile of the causative organisms in patients who failed treatment was not significantly different from that in patients who had no complications. Staphylococcus aureus was the most common pathogen in all groups. All patients included in the study confirmed that they had taken their antibiotics regularly. Unfortunately, this could not be analyzed further because the majority of patients took antibiotics at home. This is undoubtedly a limitation of the study and should be analyzed further.

However, it is worth noting that in contrast to our study, the antibiotic regimen was not specified in many studies [20,21,22,23,24]. However, it provides transparency on antibiotic therapy in consecutive patients and shows that there may be a risk of treatment failure despite adequate antibiotic therapy.

In comparison with previously published data, we found some similarities to our analyzed results. In the literature, Hadjipavlou et al. described despite antibiotic treatment, an additional surgical treatment was required in 25–55%. In our conservative-treated cohort, 40% had treatment failure. Previous studies have shown that mechanical back pain is often the result of non-surgical treatment [25]. Hadjipaylou et al. mentioned that patients with disabling back pain treated surgically had a better clinical outcome than those treated with antibiotics alone. Residual pain in non-surgically treated patients was often due to kyphosis and pseudarthrosis. This suggests that pharmacological treatment may control the infection but does not necessarily prevent deformity [26]. Martino et al. also describe that deformity can occur as a complication of the conservative treatment of spondylodiscitis [27]. In our study, we were able to show that age, cervical localization and a high CCI are significant predictors of treatment failure after conservative therapy.

If there is no instability, conservative therapy is preferred even in the presence of osteolysis. In a retrospective case series analysis, Flamme et al. also recommended conservative therapy for patients with no indication for surgical treatment, with consistent immobilization of the patient in a cast orthosis for six weeks, followed by a further six weeks of brace treatment. The orthoses produced a kyphotic posture, resulting in rapid pain relief [9].

However, prolonged immobilization with an orthosis is also associated with high risks. In addition to the risks of bed rest, a high rate of pseudoarthrosis and instability, both of which can ultimately lead to kyphotic deformity and chronic pain syndromes, has been described (16–50%) [12,28]. We therefore did not use orthosis in patients who were treated conservatively.

Nevertheless, we were able to show in our analysis that treatment failure was detected in 40% of patients treated conservatively. Increasing osteolysis was seen in 34% of cases. Even in patients treated purely conservatively, it was found that in 13% of cases, the patients continued to suffer from pain and, in line with this, there was a morphological change in the spine. Finally, we were able to show in our analysis that localization, age and a high CCI are associated with treatment failure.

The CCI score predicts 10-year survival in patients with multiple comorbidities. It is particularly used in oncology to guide treatment decisions [29]. In our study, a significant correlation between treatment failure and a high CCI score has been shown (*p* = 0.001). This can be used as a key factor in treatment decisions, especially for patients with no criteria for surgical treatment, to avoid potential treatment failure. However, it is also important to remember that a high CCI is associated with a high mortality rate. It is certainly advantageous to individualize the decision in such patients.

In our study, the mean follow-up time was 37.1 ± 31.8 (range 6–96 months). In the operative cohort, all included patients had a good outcome and demonstrated clinical healing of the spinal infection. The patients no longer described back pain. Only in 28 cases (21.9%) did patients describe intermittent back pain that does not restrict their everyday life. There was no evidence of infection. Bony fusion was achieved in 97.7% of cases, with no evidence of loosening or breakage of internal fixation. In the literature, similar results are described [11,30]. The 30 patients who underwent initial conservative treatment followed by surgery were very satisfied with the clinical outcome. A total of 12.5% reported mild back pain that did not interfere with daily activities. CT showed no more deformity or increase in osteolysis. The only conservative-treated-group (52 patients) also demonstrated clinical healing of the spinal infection. This cohort was only treated conservatively. Secondary surgery was not performed. Nevertheless, from these 52 patients, 33 (63%) described residual back pain. For 14 (42.4) of these 33 patients, the CT scan showed the onset of kyphosis. The initial CT scan, which was performed at the time of diagnosis, showed no deformity. In summary, it can be said that early surgical treatment also had a good outcome in the long-term follow-up. Also, Thavarajasingam et al. described in a meta-analysis with an overall pooled sample size of 10,954 patients that early surgical management may be more effective than conservative therapy for spondylodiscitis, and is associated with a 40% risk reduction in relapse/failure and a 39% risk reduction in mortality [15]. Thavarajasingam et al. also described that no studies included in their study reported that conservative treatment had superior clinical outcomes. This agrees with our results. Early surgical treatment resulted in superior neurological outcomes [1].

With this study, we were able to show that these predictors can help to recognize a possible treatment failure at an early stage and to make the treatment decision based on the patient and thus avoid a standardized procedure without considering the overall situation of the patient. In particular, this can also prevent secondary deformity from occurring and thus avoid more extensive and costly surgery with a significantly increased risk. This may be an important step in the decision-making process.

## 5. Conclusions

In our study, we were able to show that factors such as age, cervical spine and a high CC index were significantly associated with treatment failure. This was due to increasing bone destruction. Early surgical treatment may prevent the progression of osteolysis and long-term complications such as persistent back pain and the need for subsequent complex surgery. These predictors may also help guide individualized, patient-specific therapy for conservatively treated patients, thereby improving treatment outcomes.

### Limitation

This study is limited by its retrospective design. Whether sufficient antibiotic therapy was administered at home cannot be verified. This is a significant limitation and should be noted. Another limitation is possible selection bias or variable follow-up periods that might have influenced the results. Further prospective data are also needed to evaluate this finding in a larger cohort of patients. Another limitation is that complications were not analyzed separately for different anatomical sites. The amount of deformation was not taken into account.

## Figures and Tables

**Table 1:** Baseline demographic and clinical characteristics of patients treated conservatively.

Demographic Data	
**Conservative-Treated Cohort**	**n = 86 (35.1)**
Female	24 (28.4)
Male	62 (71.6)
Mean age, yrs	65.42
Affected level	
1	75 (88.3)
2	8 (9.3)
>2	3 (2.4)
Charlson Comorbidity Index (CCI)	
0	5 (5.8)
1	3 (3.5)
2	10 (11.6)
3	14 (16.3)
4	21 (24.4)
5	33 (38.4)
Symptoms and signs	
Sensory deficit	5 (5.8)
Motor weakness	3 (3.5)
Back pain	85 (98.8)
Radicular pain	11 (12.8)
Affected level	
1	67 (78)
2	12 (14)
>2	7 (8)
Location	
Cervical/cervicothorakal	8 (9.3)
Thoracal	17 (19.8)
Thoracolumbar	9 (10.5)
Lumbar/lumbosacral	52 (60.5)
Treatment failure	34 (40)
Extent of osteolysis after antibiotic therapy	31.9%

**Table 2:** Baseline demographic and clinical characteristics of patients treated surgically.

Demographic Data	
**Operative-Treated Cohort**	**n = 159 (64.9)**
Female	39 (24.5)
Male	120 (75.5)
Mean age, yrs	64.7
Affected level	
1	108 (67.9)
2	35 (22.0)
>2	16 (10.1)
Charlson Comorbidity Index	
0	7 (4.4)
1	15 (9.4)
2	28 (17.6)
3	41 (25.8)
4	47 (29.6)
5	21 (13.2)
Symptoms and signs	
Sensory deficit	24 (15.1)
Motor weakness	29 (18.2)
Back pain	159 (100)
Radicular pain	96 (60.4)
Affected level	
1	113 (71.1)
2	32 (20.1)
>2	14 (8.8)
Location	
Cervical/cervicothorakal	38 (23.9)
Thoracal	31 (19.5)
Thoracolumbar	21(13.2)
Lumbar/lumbosacral	69 (43.4)
Treatment failure	6 (4)
Extent of osteolysis after surgery	3.8%

**Table 3:** Microbiological findings and pathogen distribution among patients.

Organism Isolated	n = 245 (%)
*Staphylococcus aureus*	137 (55.8)
*Other staphylococcus species*	48 (19.7)
*Other coagulase negative streptococci*	33 (13.5)
*Culture negative*	27 (11.0)

**Table 4:** Summary of microbiological findings for both groups.

	Conservative n = 86 (%)	Operatively n = 159 (%)
*Staph aureus*	40 (46.5)	97 (61)
*Other staphylococcus species*	21 (24.4)	27 (17))
*Other coagulase negative streptococci*	16 (18.6)	17 (10.7)
*Culture negative*	9 (10.5)	18 (11.3)

**Table 5:** Summary of treatment failure rates and osteolysis progression in both groups.

	Conservative n = 34 (%)	Operatively n = 159 (%)
Symptoms		
Increasing back pain	34 (100)	3 (1.9)
New neurological deficits	4 (12)	2 (1.3)
Extent of osteolysis (%)	(31.9)	(3.8)

**Table 6:** Cohort characteristics, stratified by conservative and surgical treatment.

	Conservative	Operative	*p*-Value
N	86	159	
Median age in yrs	64.2	64.7	0.007
Sex, male, no. (%)	62 (71.6)	120 (75.5)	0.8
Affected level 1, no. (%)	75 (88.3)	113 (71.1)	0.3
Affected level 2, no. (%)	8 (9.3)	32 (20.1)	0.06
Affected level > 2, no. (%)	3 (2.4)	14 (8.8)	0.14
CCI 0, no. (%)	5 (5.8)	7 (4.4)	0.6
CCI 1, no. (%)	3 (3.5)	15 (9.4)	0.1
CCI 2, no. (%)	10 (11.6)	28 (17.6)	0.3
CCI 3, no. (%)	14 (16.3)	41 (25.8)	0.17
CCI 4, no. (%)	21 (24.4)	47 (29.6)	0.5
CCI 5, no. (%)	33 (38.4)	21 (13.2)	<0.001
Cervical/cervicothorakal no. (%)	8 (9.3)	38 (23.9)	0.018
Thoracal no. (%)	17 (19.8)	31 (19.5)	1.0
Thoracolumbar no. (%)	9 (10.5)	21 (13.2)	0.6
Lumbosacral no. (%)	52 (60.5)	69 (43.4)	0.1

## Data Availability

The raw data supporting the conclusions of this article will be made available by the authors, without undue reservation.
